# Mild behavioral impairment correlates of cognitive impairments in older adults without dementia: mediation by amyloid pathology

**DOI:** 10.1038/s41398-021-01675-2

**Published:** 2021-11-10

**Authors:** Yan Sun, Wei Xu, Ke-Liang Chen, Xue-Ning Shen, Lan Tan, Jin-Tai Yu, for the Alzheimer’s Disease Neuroimaging Initiative

**Affiliations:** 1 Department of Neurology, Qingdao Municipal Hospital, Qingdao University, Qingdao, China; 2 Department of Neurology and Institute of Neurology, Huashan Hospital, State Key Laboratory of Medical Neurobiology and MOE Frontiers Center for Brain Science, Shanghai Medical College, Fudan University, Shanghai, China

**Keywords:** Learning and memory, Human behaviour

## Abstract

The relationship between mild behavioral impairment (MBI) and Alzheimer’s disease (AD) is intricate and still not well investigated. The purpose of the study is to examine the roles of the AD imaging pathologies in modulating the associations of MBI with cognitive impairments. We analyzed 1129 participants (563 [49.86%] female), who had measures of Neuropsychiatric Inventory Questionnaire (NPI-Q), cognition, and amyloid PET AD biomarkers from the Alzheimer’s disease Neuroimaging Initiative (ADNI). We assess the longitudinal neuropathological and clinical correlates of baseline MBI via linear mixed effects and Cox proportional hazard models. The mediation analyses were used to test the mediation effects of AD pathologies on cognition. We found that MBI was associated with worse global cognition as represented by Mini-Mental State Examination (MMSE) (*p* < 0.001), and higher β-amyloid burden (*p* < 0.001). β-amyloid partially mediated the effects of MBI on cognition with the mediation percentage varied from 14.67 to 40.86% for general cognition, memory, executive, and language functions for non-dementia individuals. However, no significant associations were discovered between MBI and tau burden or neurodegeneration. Furthermore, longitudinal analyses revealed that individuals with MBI had a faster increase in brain amyloid burden (*p* < 0.001) and a higher risk of clinical conversion (HR = 2.42, 95% CI = 1.45 to 4.01 *p* < 0.001). In conclusion, MBI could be an imperative prediction indicator of clinical and pathological progression. In addition, amyloid pathologies might partially mediate the influences of MBI on cognitive impairments and AD risk.

## Introduction

Characterized by abnormal amyloid deposition, tau phosphorylation and neurodegeneration in pathology and cognitive and behavioral impairments in clinic, Alzheimer’s disease (AD) has a prolonged asymptomatic phase in the course of the Alzheimer’s continuum before pathological biomarkers appeared [1–4]. Selecting individuals who are at high risk of suffering AD is the critical step to give effect to accurate diagnosis and timely intervention at the early preclinical and prodromal stages of AD and drug development [5]. Neuropsychiatric symptoms (NPSs) mainly manifested as disturbances of mood, perception, and behavior linked with neurodegenerative disease, which are regarded as noncognitive or behavioral and psychiatric symptoms of dementia [6]. NPSs occurred in the prodromal or mild cognitive impairment (MCI) stages of dementia and associated with an increased risk of AD and pathologically associated with greater plaque and tangle burden indicating that preventing NPSs could be a promising way for early intervention [7–9].

The ISTAART-AA criteria for mild behavioral impairment (MBI) has been developed by the International Society to Advance Alzheimer’s Research and Treatment (ISTAART) NPS Professional Interest Area to promote investigation into the correlation between NPSs and dementia [1]. MBI characterized by the new emerged and sustained NPSs may be an early manifestation of neurodegenerative disease. The ISTAART-AA MBI criteria emphasize the importance of a significant change from the person’s usual behavior or personality persisting for at least 6 months in the following domains: absence of drive and motivation; emotional dysregulation; impulse dyscontrol; social inappropriateness; and abnormal perception and thought content, assessed individually and collectively for their impact on cognition. In addition, psychiatric illness was explicitly excluded a priori. MBI is easy to capture using several simple rated scales for NPSs [10]. For instance, the Neuropsychiatric Inventory Questionnaire (NPI-Q) could reflect NPI items into MBI domains. MBI therefore provides a flexible and convenient method for selecting a population at higher risk for cognitive decline and dementia.

Previous studies have demontrated that MBI was a marker of cognitive decline in intact-elder individuals [11, 12] and also associated with AD pathology [13]. However, although MBI and amyloid burden were revealed to contribute to cognitive decline independently, it is still disputable whether amyloid could modulate the relationships of MBI with cognitive functions. In the current study, we hypothesis that the effects of MBI on cognition may be modulated by AD pathology in non-dementia elderly individuals. Herein, we test the hypothesis by evaluating the relationships between the MBI scores, AD imaging biomarkers including brain burden of β-amyloid, brain burden of tau and neurodegeneration, and cognitive impairment in participants without dementia. Detecting sensitive and specific markers of very early AD progression is proposed to help develop new treatments and to decrease the time and cost of clinical trials [14].

## Materials and methods

### Subjects

Data were obtained from the ADNI database (http://adni.loni.usc.edu). The multicenter ADNI project is designed to predict the early onset of AD through investigating clinical, imaging, genetic, and biochemical biomarkers and have been recruited participants from more than 50 sites across the United States and Canada. The participants are older adults aged 55–90 years and their cognitive trajectories and the biomarkers were repeatedly assessed during the follow-up period to track the pathology as the disease progressed. Written informed consent was obtained on human experimentation at each institution and detailed information can be found at http://adni.loni.usc.edu/study-design.

In the study, we selected 1129 participants without dementia, including 543 MCI and 586 CN individuals. The population included ADNI-1 and ADNI-2 and GO participants enrolled into the MBI > 0 and MBI = 0 cohorts, were tested for positron emission tomography (PET) and NPI-Q.

### PET imaging measures

All PET data used were from the UC Berkeley and Lawrence Berkeley National Laboratory. Florbetapir (AV45) SUVRs calculated by averaging across four cortical regions (frontal, anterior/posterior cingulate, lateral parietal, lateral temporal) were used for brain amyloid burden, and then divided by the whole cerebellum as reference region. Brain tau deposit used a composite metaROI was measured via the flortaucipir (AV-1451) processing method and the bilateral entorhinal, amygdala, fusiform, inferior, and middle temporal regions were considered for tau-PET assessment [15]. Brain neurodegeneration used hypometabolism assessed by 18F-fluorodeoxyglucose (FDG) PET, which was from the average of five metaROIs (left angular gyrus, right angular gyrus, bilateral posterior cingular, left inferior temporal gyrus, right inferior temporal gyrus) [16].

### Assessment of mild behavioral impairment

The MBI checklist (MBI-C), which was ascertainable instrument for MBI, was not yet incorporated into ADNI [10]. Thus, according to a published algorithm, a transformation of NPI-Q scores was approximated to MBI status [17]. Ten NPSs domains from the NPI-Q were subdivided into five ISTAART-AA MBI domains of the absence of drive and motivation (NPI-Q apathy), emotional dysregulation (NPI-Q depression/anxiety/euphoria), impulse dyscontrol (NPI-Q agression/irritability/aberrant motor behavior), social inappropriateness (NPI-Q disinhibition), and abnormal perception and thought content (NPI-Q delusions/hallucinations). The ISTAART-AA MBI criteria put an emphasis on the necessary of a distinct change from the person’s usual behavior or personality persisting for at least 6 months in the five domains [1, 13]. Finally, adding together the five transformed domains to obtain the approximated MBI status. Participants with an MBI score >1 were considered as MBI+ status and participants with an MBI score of 0 were considered as MBI– status [18]. All individuals were over the age of 55 and none of them met criteria for any major neuropsychiatric disorder.

### Cognitive assessment

We used multiple scales to assess cognitive functions, including the global cognition by Mini-Mental State Examination (MMSE), the Montreal Cognitive Assessment Scale (MoCA) and the cognitive section of Alzheimer’s Disease Assessment Scale (ADAS). Participants also underwent neuropsychological evaluation of three cognitive domains (executive, memory and language functions), which were assessed by reviewing the neuropsychological batteries to identify items that could be considered indicators of these three domains [19, 20]. Above mentioned scales were used to represent general and specific cognition functions.

### Statistical analyses

Data were presented mean (standard deviation, SD) or number (percentage, %) when appropriate. First, we examined the relationships of MBI scores with cognition and AD PET imaging biomarkers in different groups. Linear regression models were used to investigate the cross-sectional relationships of MBI scores with PET imaging biomarkers and cognitive measures. Then, the relation between MBI and clinical progression was tested by calculating cumulative incidence using the Kaplan–Meier method. Clinical progression was defined as: (1) diagnosed as MCI/dementia for baseline CN individuals, (2) diagnosed as dementia for baseline MCI individuals. Cox proportion hazards model was conducted to estimate the hazard ratio (HR) with 95% confidence interval (CI). Individuals who did not develop MCI/AD or who were lost were censored at the time of their last evaluation.

Next, the mediation analyses were used to evaluate whether the association between MBI and cognition was mediated amyloid pathologies. Baron and Kenny have proposed the calculation methods through linear regression models fitting [21] and we also reference the detail methods in an previous study [22]. The first equation regressed mediator (PET imaging biomarkers) on the independent variable (MBI). The second equation regressed the dependent variable (cognitive scores) on the independent variable. The third equation regressed the dependent variable on both the independent variable and the mediator variable. Diagnosis was not considered as a covariate due to the close relationship between cognitive score and clinical diagnosis (MCI vs CN) in the analyses with cognitive scores as the dependent variables. The establishment of the mediation effects must satisfy the following criteria simultaneously: (1) MBI was associated with PET imaging biomarkers significantly; (2) MBI was associated with cognitive measures significantly; (3) PET imaging biomarkers were associated with cognitive measures significantly; (4) the association between MBI and cognition was diminished when PET imaging biomarkers (the mediator) were added in the regression model. In all above analyses, age, gender, diagnosis, education, and *APOE*4 status were controlled.

Finally, we used the linear mixed effects models to track the longitudinal relationship between MBI and the imaging biomarkers. The PET imaging biomarkers were considered as the dependent variable and MBI total scores and MBI status (MBI scores >1 considered as MBI+ while MBI scores = 0 considered as MBI−) were treated as independent variables. The linear mixed effects models included random intercepts and slopes for time and an unstructured covariance matrix for the random effects, and regarded the interaction between time (continuous) and the dependent variable (MBI + vs MBI- or MBI total scores) as a predictor. We have adjusted age, gender, education, diagnosis and *APOE*4 status as covariates in all analyses.

All statistical analyses and figure preparation were conducted on R software (version 3.5.1). All above analyses were conducted by “lme4”, “survival”, “ggplot2”, “ggpubr”, “magrittr”, “survminer”, “nlme”, “car” and “mediate” packages in R 3.5.1 software. Two-sided *P* value < 0.05 were considered as significance.

## Results

### Characteristics of participants

A total of 1129 individuals without dementia (543 MCI and 586 CN) were included into the study. The mean (SD) age of the MBI = 0 cohorts was 72.35 (6.25) years old and females accounted for 53.89%. MBI > 0 cohorts have higher proportion of male, *APOE*4 positive and younger with mean age of 71.68 (7.25), and they had poorer baseline global cognition scores and higher burden of cerebral amyloid burden. Characteristics of participants grouped by baseline diagnosis were summarized in Table 1.

**Table 1:** Baseline characteristics of participants according to groups.

	Non-AD			MCI			CN		
	MBI > 0	MBI = 0	*p* value	MBI > 0	MBI = 0	*p* value	MBI > 0	MBI = 0	*p* value
No.	*n* = 385	*n* = 744		*n* = 296	*n* = 247		*n* = 89	*n* = 497	
Age	71.68 ± 7.25	72.35 ± 6.25	0.032	71.67 ± 7.57	72.48 ± 7.18	0.026	71.71 ± 6.07	72.23 ± 6.25	0.215
Education (year)	16 (14–18)	16 (15–18)	0.456	16 (14–18)	16 (14–18)	0.444	16 (15–18)	17 (15–18)	0.654
Female	162 (42.07%)	401 (53.89%)	0.321	110 (37.16%)	121 (48.98%)	0.219	52 (58.42%)	279 (56.13%)	0.341
*APOE* ɛ4 carriers	170 (44.15%)	267 (35.88%)	0.004	145 (48.98%)	111 (44.93%)	0.013	25 (28.08%)	156 (31.38%)	0.134
Florbetapir	*n* = 349 1.206 ± 0.231	*n* = 591 1.147 ± 0.198	0.002	*n* = 275 1.238 ± 0.236	*n* = 217 1.201 ± 0.222	0.044	*n* = 74 1.087 ± 0.168	*n* = 374 1.116 ± 0.176	0.606
Flortaucipir	*n* = 162 1.198 ± 0.158	*n* = 378 1.188 ± 0.168	0.125	*n* = 87 1.236 ± 0.205	*n* = 79 1.260 ± 0.246	0.146	*n* = 75 1.150 ± 0.096	*n* = 299 1.163 ± 0.136	0.067
FDG	*n* = 353 1.256 ± 0.131	*n* = 524 1.291 ± 0.123	0.769	*n* = 281 1.243 ± 0.129	*n* = 240 1.270 ± 0.135	0.654	*n* = 72 1.308 ± 0.128	*n* = 284 1.310 ± 0.108	0.089
ADAS	*n* = 339 9.18 ± 5.17	*n* = 588 7.40 ± 3.72	<0.001	*n* = 265 9.95 ± 5.27	*n* = 216 9.18 ± 4.06	0.222	*n* = 74 6.31 ± 3.56	*n* = 372 6.37 ± 3.07	0.688
MMSE	*n* = 339 28.05 ± 2.05	*n* = 591 28.68 ± 1.47	<0.001	*n* = 265 27.80 ± 2.15	*n* = 217 28.08 ± 1.68	0.044	*n* = 74 28.97 ± 1.28	*n* = 374 29.02 ± 1.21	0.280

*No.* number of participants, *MBI* mild behavioral impairment, *APOE* ɛ4 apolipoprotein E gene, *Non-AD* participants without alzheimer’s disease, *MCI* mild cognition impairment, *CN* cognitively normal, *FDG* 18F-fluorodeoxyglucose, *ADAS* Alzheimer’s disease assessment scale, *MMSE* mini-mental state examination, *MoCA* Montreal cognitive scale.

### Associations of MBI with PET imaging biomarkers and cognitive measures

As Table 2 summarized, in the cross-section, individuals with higher MBI total scores which means more NPSs had greater cerebral amyloid deposition, as indicated by higher β-amyloid (*β* = 0.018, *p* = 0.006) burden and lower FDG PET (*β* = −0.020, *p* < 0.001) in older individuals without dementia. The relationship reminded significant after controlling for age, gender, years of education, diagnosis (in total samples), and *APOE*4 status. When categorized the total sample into two subgroups, there is no significant findings except the lower FDG PET in MCI group. Also, no significant associations with tau PET were found in all groups. In addition, non-AD individuals with higher MBI scores had lower global cognitive measures as represented by MMSE (*β* = −0.113, *p* = 0.037), MoCA (*β* = −0.508, *p* < 0.001), ADAS (*β* = 0.619, *p* < 0.001) (Supplementary Table 1).

**Table 2:** Cross-section and longitudinal regressions of MBI and PET pathology according to groups.

	Non-AD individuals						CN individuals						MCI individuals					
	Aβ-PET		Tau-PET		FDG-PET		Aβ-PET		Tau-PET		FDG-PET		Aβ-PET		Tau-PET		FDG-PET	
	*β*	*p*	*β*	*p*	*β*	*p*	*β*	*p*	*β*	*p*	*β*	*p*	*β*	*p*	*β*	*p*	*β*	*p*
Cross section	*n* = 941		*n* = 702		*n* = 877		*n* = 449		*n* = 531		*n* = 356		*n* = 492		*n* = 171		*n* = 521	
MBI	**0.018**	**0.006**	0.007	0.306	**−0.020**	**<0.001**	−0.02	0.069	−0.005	0.507	−0.008	0.363	0.143	0.098	0.003	0.823	**−0.011**	**0.028**
Age	**0.007**	**<0.001**	0.001	0.122	**−0.003**	**<0.001**	**0.06**	**<0.001**	**0.003**	**<0.001**	**−0.003**	**<0.001**	**0.008**	**<0.001**	−0.001	0.698	−**0.004**	**<0.001**
Education (year)	−0.001	0.604	−0.001	0.532	0.001	0.337	−0.002	0.392	0.003	0.173	0.002	0.295	0.001	0.642	−0.004	0.487	<0.001	0.757
Female	**−0.032**	**0.012**	**−0.036**	**0.004**	**−0.020**	**0.030**	**−0.061**	**<0.001**	**−0.044**	**<0.001**	−0.011	0.326	**−0.038**	**0.047**	−0.06	0.082	−0.019	0.096
*APOE* ɛ4	**0.142**	**<0.001**	**0.073**	**<0.001**	**−0.034**	**<0.001**	**0.103**	**<0.001**	**0.028**	**0.011**	−0.008	0.458	**0.150**	**<0.001**	**0.094**	**<0.001**	**−0.040**	**<0.001**
LME model	*N* = 633		*N* = 174		*N* = 360		*N* = 300		*N* = 104		*N* = 151		*N* = 333		*N* = 70		*N* = 209	
MBI	**0.025**	**<0.001**	−0.006	0.734	0.007	0.235	0.002	0.868	**0.025**	**0.032**	−0.001	0.752	**0.020**	**0.014**	−0.016	0.477	−0.001	0.628
MBI*TIME	**0.004**	**<0.001**	−0.002	0.498	−0.003	0.057	**0.004**	**0.001**	−0.003	0.142	−0.002	0.727	**0.002**	**0.048**	−0.004	0.506	−0.008	0.115

Effect sizes are calculated as standardized betas (*β*). Significant effects (*P* < 0.05) are shown in bold. Cross section, linear regressions were used to explore the relationships of baseline MBI scores with PET imaging biomarkers. LME model, linear mixed effects models were used to explore the relationship between baseline MBI scores and the progression of PET imaging biomarkers.

*MBI* mild behavioral impairment, *ApoE* apolipoprotein E gene, *Non-AD* participants without Alzheimer’s disease, *MCI* mild cognition impairment, *CN* cognitively normal, *APOE* ɛ4 apolipoprotein E gene.

### Causal mediation analyses

**Fig. 1:**
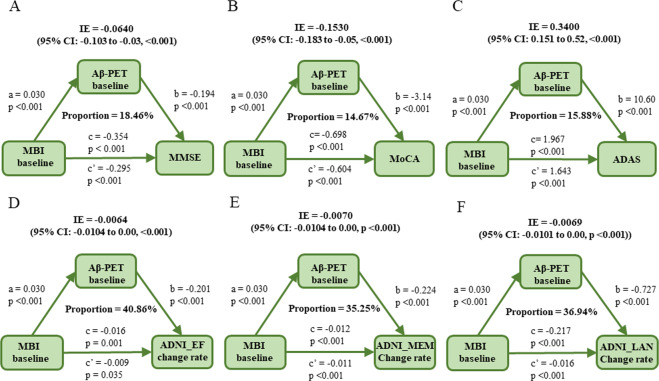
Mediation analyses with cognitive domains, MMSE, MoCA, and ADAS as cognitive outcomes in non-dementia individuals. The relationship between MBI and global cognition measured by MMSE (**A**), MoCA (**B**), ADAS (**C**), as well as cognitive domain of executive (**D**), memory (**E**) and language (**F**) function was mediated by β-amyloid. IE indirect effect. Path a, regression model of β-amyloid on MBI. Path b, regression model of the cognitive scores on β-amyloid. Path c, regression model of cognitive scores on MBI. Path c′, regression model of cognitive scores on both MBI and β-amyloid.

All the above findings suggested that MBI was not only an independent risk factor for cognition decline, but also associated with amyloid pathology. To examine whether amyloid pathology was a potential modulator of MBI on cognition, we further conducted mediation analyses. As the results showed, the relationship between MBI and cognitive impairment was mediated mainly by β-amyloid (Fig. 1). In the total samples, in the first equation, MBI was significantly associated with higher levels of β-amyloid deposition (*p* = 0.033). In the second regression, MBI showed a significant association with poorer global cognition measured by MMSE (*p* < 0.001), MoCA (*p* < 0.001), and ADAS (*p* < 0.001). Finally, in the third equation, when put the amyloid indicator and MBI simultaneously into the model, we found that the influences of MBI on global cognition remained but were significantly diminished. The effect was considered partial mediation with the proportion of mediation varying from 14.67% to 18.46%. The change rates of cognitive domains (memory, executive and language functions) were also calculated. Similar results were concluded for the three functional domains. The proportion of mediation varied from 35.25% to 40.86% (*p* < 0.05). These findings further supported the hypothesis that amyloid pathology could at least partially modulate the influences of NPSs on cognitive functions.

When stratified all individuals into MCI or CN groups, the mediation effects were not established due to criteria were not simultaneously satisfied (Supplementary Figs. 1 and 2). In MCI group, interestingly, the mediation effects were not qualified, because the second criteria (MBI was associated with cognitive measures significantly) was not reached through the indirect effects calculated in the model were all significant. That probably due to the clinical diagnosis (MCI or CN) were intimate to cognitive scores (MMSE, MoCA etc.), stratifying participants into groups according to diagnosis weaken the relationship between independent variable (MBI) and cognitive scores.

As mentioned above, given the lack of associations between MBI and tau burden and FDG, the mediation relationships of the two AD biomarkers between MBI and cognitive impairments have not established in the present study (Supplementary Figs. 3 and 4).

### Longitudinal relationship between MBI and amyloid pathology

**Fig. 2:**
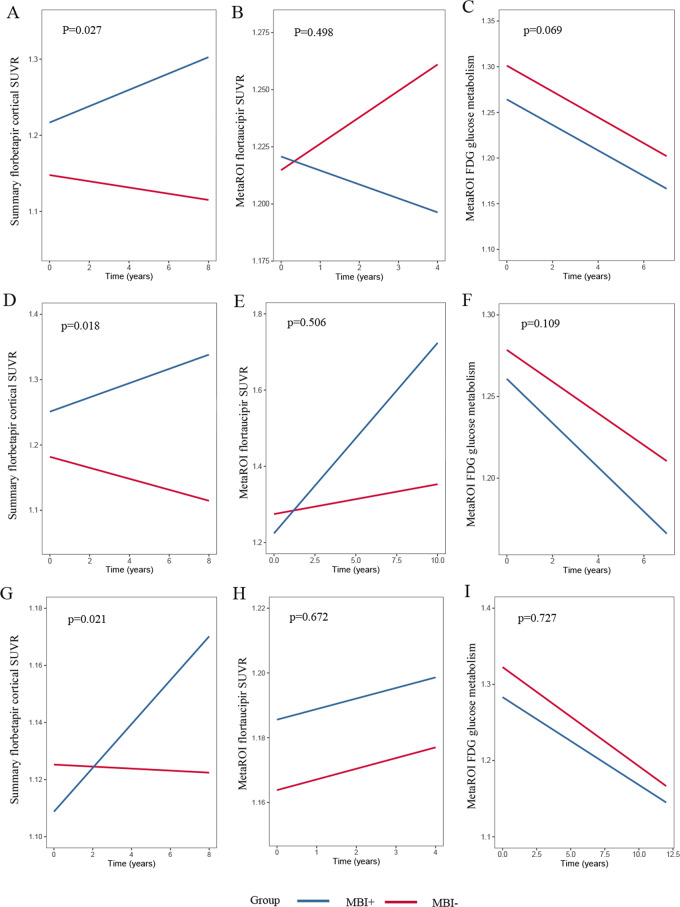
Longitudinal changes of amyloid, tau, and neurodegeneration according to MBI status. **ABC** Longitudinal changes of β-amyloid, tau, and neurodegeneration in non-AD individuals; **DEF** Longitudinal changes of β-amyloid, tau, and neurodegeneration in MCI individuals; **GHI** Longitudinal changes of β-amyloid, tau, and neurodegeneration in CN individuals.

From total samples, numbers of individuals who provided the necessary follow-up PET imaging data were detailed in Table 2 and the range and distribution of MBI scores were also detailed (Supplementary Table 2). In the longitudinal section, controlling for age, sex, education, *APOE*4 status, clinical diagnosis, participants with higher MBI total scores predicted higher β-amyloid deposition in total samples (*n* = 633, *β* = 0.003, interaction with time *p* < 0.001). We also found that higher MBI scores displayed faster elevation of β-amyloid burden in both MCI (*n* = 300, *β* = 0.004, interaction with time *p* = 0.001) and CN (*n* = 333, *β* = 0.002, interaction with time *p* = 0.048) groups. Then we categorized the total samples into MBI+ and MBI− groups. Participants with a baseline MBI total score of 1 were excluded from the study due to diagnostic uncertainty [18]. As shown in Fig. 2 the burden of cerebral β-amyloid deposition was significantly higher in MBI+ group compared with individuals of MBI− status, which indicated the trends of greater annual accumulating rates. Same findings were observed in CN and MCI groups. In the present study, no relationships were established in the tau PET and FDG PET.

### MBI and incident AD risk

**Fig. 3:**
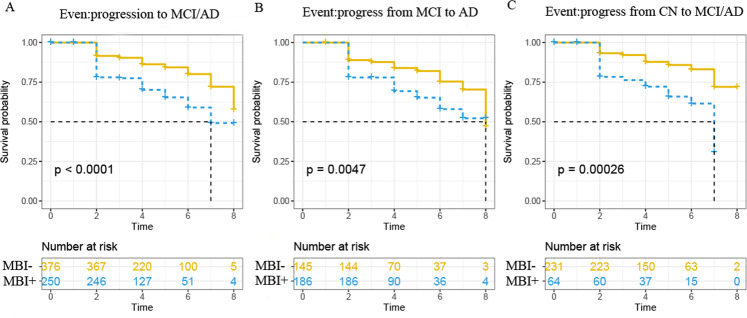
Kaplan–Meier curves showing survival probability of clinical progression according to groups. **A** Progression from CN to MCI and progression from MCI to AD dementia. **B** Progression from MCI to AD dementia. **C** Progression from CN to MCI or AD dementia.

The survival curves of MBI status and clinical progression were calculated in participants who provided β-amyloid data and were exhibited in Fig. 3. Individuals MBI+ group showed higher conversion risk (Supplementary Table 3) in clinical progression, based on MBI− group. In CN group, individuals with MBI+ presented a progression rate to MCI/AD dementia of 29.68% while MBI- individuals presented 12.55% (HR = 2.81, 95% CI = 1.59–4.96, *p* < 0.001). As the same, MBI+ individuals (31.18%) showed a higher progression rate to AD dementia compared with MBI− individuals (17.24%) in MCI group (HR = 1.93, 95% CI = 1.21 to 3.06, *p* = 0.005).

## Discussion

The present study provided several lines of evidence for the associations of baseline MBI with Alzheimer’s pathologies and clinical progression among non-dementia individuals. Furthermore, it found that the influence of MBI on cognition was partially mediated by amyloid pathology. As the results showed, individuals with MBI possessed a faster accumulation of brain amyloidosis and a higher risk of clinical conversion, suggesting a predictive link of MBI with amyloidosis progression. In addition, higher MBI scores, as reflected by serious NPSs, predicted a more severe future progress. All these findings support that MBI can provide imperative information for early detection and intervention. Base on the above findings, it could be inferred that cerebral amyloid deposition could mediate the effects of MBI on cognitive impairment.

The present study further verified that the predictive relationship of baseline MBI in non-dementia individuals with the progress of amyloid pathology and firstly revealed the relationship of MBI and cognitive impairment may via amyloid pathology. It crucially replenishes the gap in current research and further elaborates on the impact of NPSs on subsequent pathological and clinical progression. The previous study also suggested that drugs either reducing amyloid, preventing tau hyperphosphorylation, or modifying neurodegeneration were reliable for earlier intervention through feasible methods for early detection of preclinical AD [13].

MBI has been identified to be indicators of preclinical AD previously [2, 12, 23]. A recent review about the role of NPSs in diagnostic criteria for AD dementia has explained that MBI is the harbinger of a progressive dementia syndrome occurred with MCI or not [24]. It was reported as a representation of the neurobehavioral axis of pre-dementia risk states and complemented the neurocognitive axis represented by subjective cognitive decline and MCI. Actually, clinical and imaging biomarker studies have generally accepted that MBI is a risk state and marker of early disease [18]. Previous research has validated MBI as a preclinical dementia syndrome and associated with amyloid positivity, before tau and neurodegeneration in older adults with normal cognition, which was consistent with our findings that MBI was a cause to the cognitive decline mainly via β-amyloid, also in advance of tau or neurodegeneration biomarkers, irrespective of the presence or absence of subjective cognitive complaints [13]. In addition, the relationship between dementia amyloid pathologies and NPSs have also been supported by recent studies. For instance, NPSs such as anxiety can be further investigated by staging β-amyloid deposition in both cortical and subcortical regions [25], and previous study has also reported that increased depression-anxiety scores correlated to elevated cortical amyloid deposition [26]. All these findings supposed the synergetic roles of MBI and amyloid pathology, which was consistent with the mentioned hypothesis. In the present study, we not only revealed that MBI effected cognitive function regardless of amyloid burden, but also found a mediation effect of amyloid pathology.

We did not find the influences of MBI on cognitive functions were mediated by tau or neurodegeneration. The hypothesis of the present study is that MBI was associated with early but not with later-stage AD pathophysiology [27]. Though considering that tau pathologies could lead to cognitive decline independent of amyloid, significant tau aggregation is rarely observed in cognitive intact individuals [13]. With regard to the neurodegeneration biomarker category, it is reported that early neurodegeneration related loss of biogenic amine nuclei can potentially manifest as psychiatric symptomatology [28]. The negative findings here might be explained as considering the lack of association between MBI and tau burden and the temporal ordering of AD-related pathologies. It has been reported that Aβ pathophysiological processes became abnormal first and then downstream neuronal injury biomarkers, such as tau pathology and neurodegeneration markers became abnormal later [29–31]. But interestingly, in the cross-sectional analyses, we find that higher MBI scores corresponding to lower FDG-PET, which has been detected in other studies that pathways promoting Aβ and neurodegeneration may initially occur independently [32–34]. All above implied a hierarchical ranking of Aβ biomarkers over downstream neuronal injury biomarkers, hence, our result is not unexpected for the non-dementia individuals.

The etiology of MBI is still unclear and the mechanisms by which MBI was involved in regulating amyloid pathology are not wholly explained either. Prior study reported that depression, one of the mainly symptom of the “emotional dysregulation” domain [35], could increase neuroinflammatory cytokines and disorder brain-derived neurotrophic factor by effecting hypothalamic-pituitary-adrenal axis altered gamma-amino butyric acid system and then provided a possible pathway hypothesis [36]. Another study showed that NPSs were strongly linked to 3-methoxy-4-hydroxyphenylethyleneglycol, which was the intraneuronal metabolite of norepinephrine, and p-tau, that suggesting that the locus coeruleus -norepinephrine may be pivotal to understand connections between AD pathology and behavioral deficits in AD [37]. Actually, though amyloid pathology and MBI as potential target of early intervention to prevent AD respectively, their interactions still warrant further investigation.

There are limitations in this study. First, the specificity of estimating MBI via NPI-Q was lower compared to those with the MBI-C, because the short reference range can result in poor specificity, inappropriately capturing as cases subjects with transient symptoms and reactive conditions, and therefore, resulting in an inflated prevalence estimate [17, 38]. To elevate the accuracy of this approach to capture MBI, when stratified the MBI status as categorical variable, score of 1 were excluded, it may prevent to categorize some participants as MBI+ falsely. In addition, MBI affected multi-types of dementia [1, 39] and specificity need to be of concerns. Further researches are required to detect the specificity of MBI on multi-types of dementia.

## Conclusions

In conclusion, the present study indicated that amyloid pathology was not only contributed from MBI, but also a key mediator for influences of MBI on cognitive impairments and AD risk. Examining neurobehavioral outcomes (e.g., mood, social interaction) was highly recommended to link the pathological process to clinical symptoms, which contribute to delineate a time course and depict the impact of it on functional decline prior to overt symptom onset [40, 41]. These findings supported the hypothesis that MBI represent an early manifestation of preclinical AD and could be used to help define high-risk population who are suitable for early prevention of the disease.

## Supplementary information

Suplementary files

## Data Availability

The dataset generated and analyzed in the current study is available from the corresponding author on reasonable request.
