# Supplementary material for: Mild behavioral impairment correlates of cognitive impairments in older adults without dementia: mediation by amyloid pathology
Source: Transl Psychiatry. 2021 Nov 10;11:577. doi: 10.1038/s41398-021-01675-2 (PMC8580970; doi:10.1038/s41398-021-01675-2)
Supplement: Supplementary file 1 — Suplementary files [file 41398_2021_1675_MOESM1_ESM.docx]

Catalog

**Supplementary Table 1** 1

**Supplementary Figure 1** 2

**Supplementary Figure 2** 3

**Supplementary Figure 3** 4

**Supplementary Figure 4** 5

**Supplementary Table 2** 6

**Supplementary Table 3** 7

**Supplementary Table 1 MBI associated with cognitive measures in linear regression models**

| Cognition scales | Non-AD | | MCI | | CN | |
| --- | --- | --- | --- | --- | --- | --- |
|  | β | P value | β | P value | β | P value |
| MMSE | -0.113 | 0.037 | 0.011 | 0.870 | <0.001 | 0.998 |
| MoCA | -0.508 | <0.001 | -0.201 | 0.112 | -0.238 | 0.291 |
| ADAS | 0.619 | <0.001 | 0.143 | 0.409 | -0.124 | 0.628 |
| MEM change rate | -0.012 | <0.001 | -0.006 | 0.121 | -0.004 | 0.324 |
| EF change rate | -0.012 | <0.001 | -0.008 | 0.079 | 0.001 | 0.822 |
| LAN change rate | -0.126 | <0.001 | -0.005 | 0.278 | -0.003 | 0.501 |

Non-AD, participants without alzheimer’s disease; MCI, mild cognition impairment; CN, cognitively normal; MMSE, Mini-Mental State Examination; ADAS, Alzheimer’s Disease Assessment Scale; ADAS, Alzheimer’s Disease Assessment Scale;

MEM, Memory Function; EF, Executive Function; LAN, language Function;

All models were adjusted for age, gender, education, and *APOE*4 status;

**Supplementary Figure 1 Mediation analyses with cognitive domains, MMSE, MoCA and ADAS as cognitive outcomes in MCI individuals**


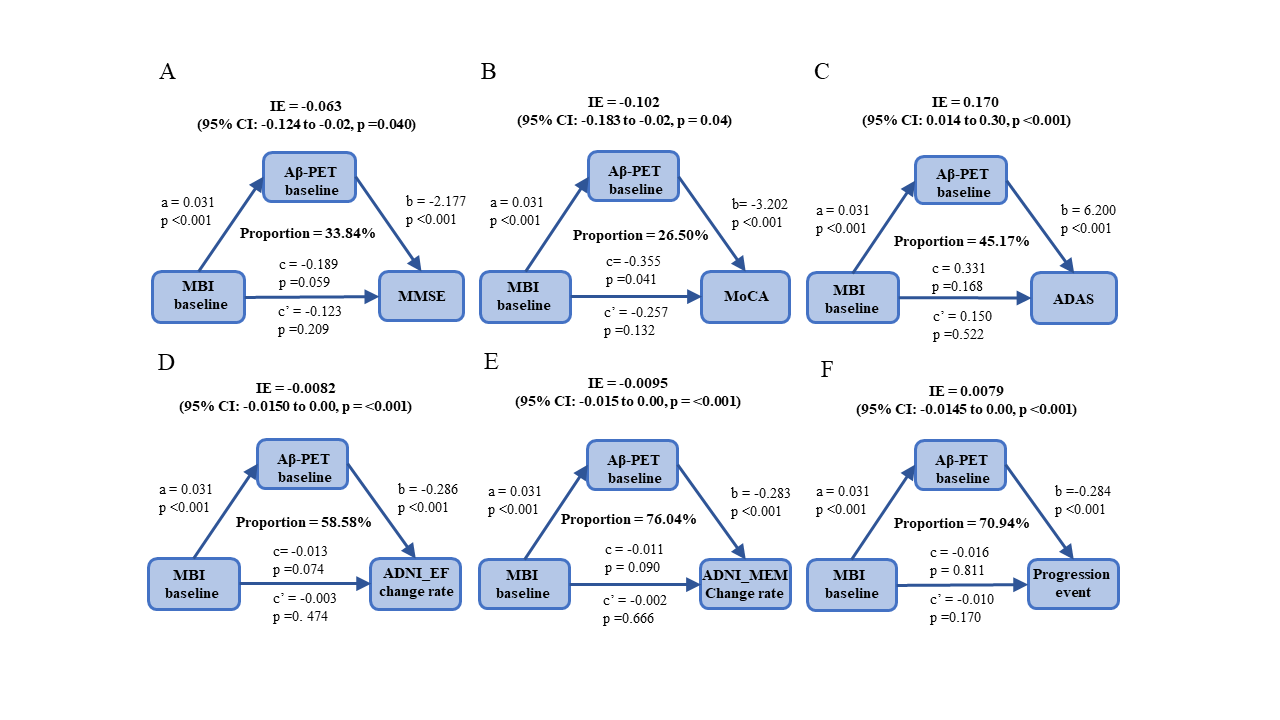


The relationship between MBI and global cognition measured by MMSE (A), MoCA (B), ADAS (C), as well as cognitive domain of executive (D), memory (E) and language (F) function was not mediated by β-amyloid.

**Supplementary Figure 2 Mediation analyses with cognitive domains, MMSE, MoCA and ADAS as cognitive outcomes in CN individuals**


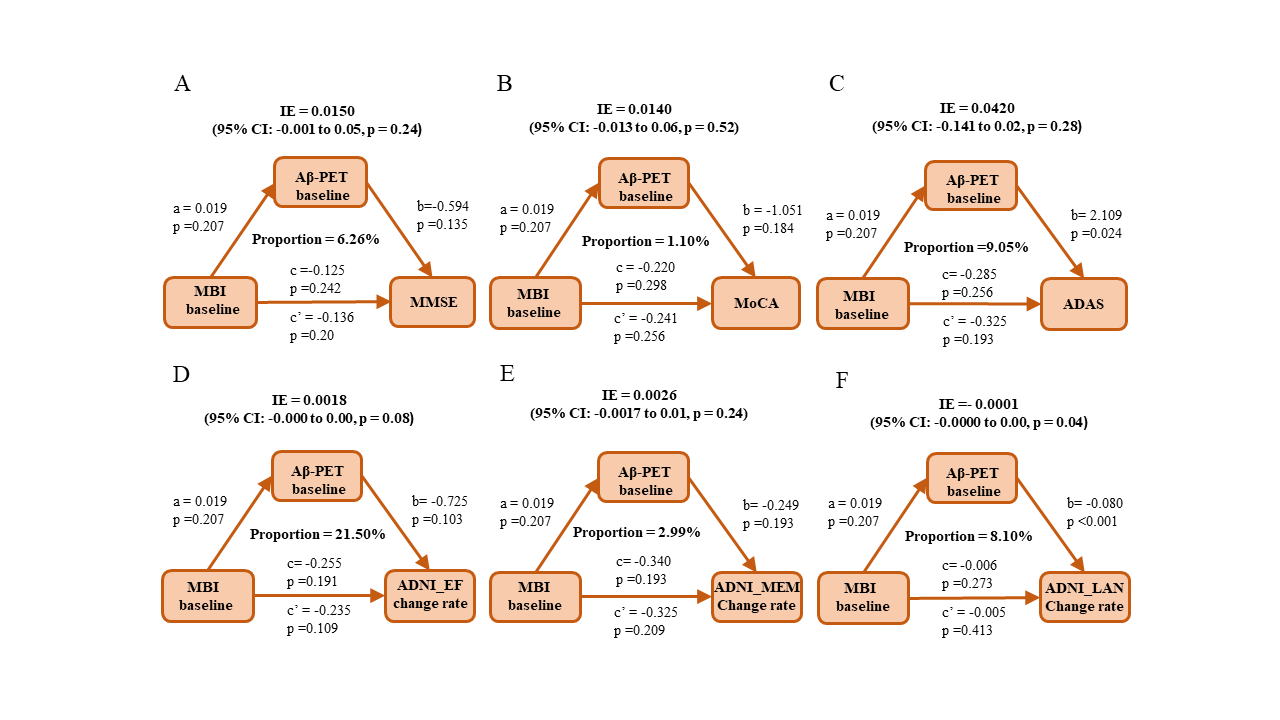


The relationship between MBI and global cognition measured by MMSE (A), MoCA (B), ADAS (C), as well as cognitive domain of executive (D), memory (E) and language (F) function was not mediated by β-amyloid.

**Supplementary Figure 3 Mediation analyses with cognitive domains, MMSE, MoCA and ADAS as cognitive outcomes in different groups**


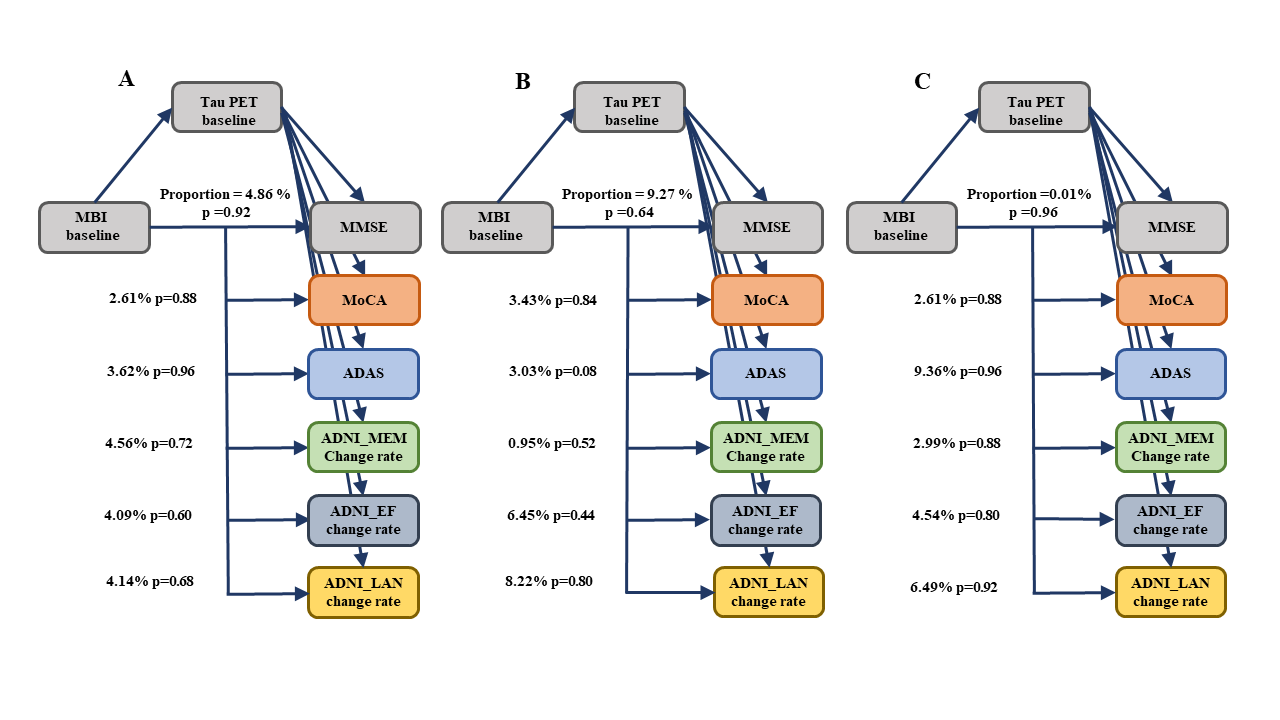


The relationship between MBI and cognitive measures, including global cognition measured by MMSE, MoCA, ADAS, memory, executive and language function was not mediated by tau pathology in non-dementia individuals (A), as well as in MCI (B) and CN (C) individuals.

**Supplementary Figure 4 Mediation analyses with cognitive domains, MMSE, MoCA and ADAS as cognitive outcomes in different groups**


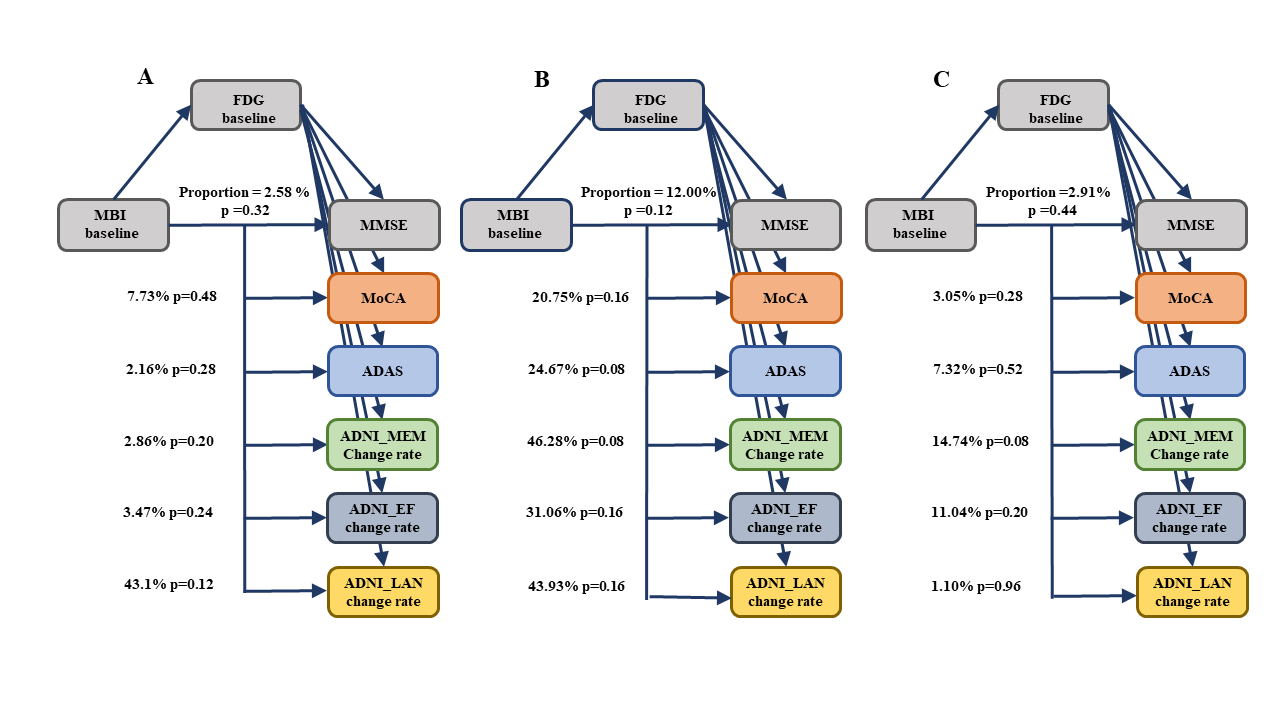


The relationship between MBI and cognitive measures, including global cognition measured by MMSE, MoCA, ADAS, memory, executive and language function was not mediated by FDG in non-dementia individuals (A), as well as in MCI (B) and CN (C) individuals.

**Supplementary Table 2 The ranges and distributions of the MBI scores in follow-up individuals**

| MBI scores | 0 | 1 | 2 | 3 | 4 |
| --- | --- | --- | --- | --- | --- |
| Aβ-PET | 383 | 144 | 62 | 40 | 4 |
| Tau-PET | 125 | 37 | 6 | 2 | 4 |
| FDG-PET | 224 | 85 | 32 | 15 | 4 |

MBI mild behavioral impairment

**Supplementary Table 3. Progression risk of MBI+ and MBI- individuals**

|  | Progression rate | Unadjusted | |  | Adjusted | |
| --- | --- | --- | --- | --- | --- | --- |
|  |  | HR (95%) | p |  | HR (95%) | p |
| Progression to MCI/AD |  |  |  |  |  |  |
| MBI- | 14.89% | ref | - |  | ref | - |
| MBI+ | 30.76% | 2.73(1.62-4.61) | <0.001 |  | 2.42(1.45-4.01) | <0.001 |
| CN to MCI/AD |  |  |  |  |  |  |
| MBI- | 12.55% | ref | - |  | ref | - |
| MBI+ | 29.68% | 3.34 (1.87-5.96) | <0.001 |  | 2.81 (1.59-4.96) | <0.001 |
| MCI to AD |  |  |  |  |  |  |
| MBI- | 17.24% | ref | - |  | ref | - |
| MBI+ | 31.18% | 2.01 (1.26-3.19) | 0.003 |  | 1.93 (1.21-3.06) | 0.005 |

Note: Hazard ratios (95% CI) calculated using Cox regression analyses, in unadjusted and adjusted models corrected

for baseline age, gender, *APOE*4 status, and years of education.
